# Malunion of the Tibia: A Systematic Review

**DOI:** 10.3390/medicina58030389

**Published:** 2022-03-05

**Authors:** Ishan Patel, Jacob Young, Austen Washington, Rahul Vaidya

**Affiliations:** 1 Department of Orthopaedic Surgery, Detroit Medical Center, Detroit, MI 48187, USA; austen.wash@gmail.com (A.W.); rahvaidya2012@gmail.com (R.V.); 2 School of Medicine, Wayne State University, Detroit, MI 48201, USA; fi3680@wayne.edu

**Keywords:** tibia, tibial, malunion, approach, outcomes, plateau, shaft, pilon

## Abstract

*Background and Objectives*: Tibial malunions are defined as tibial fractures that have healed in a clinically unacceptable position, resulting in deformity such as shortening, lengthening, abnormal rotation, or angulation. These deformities can have adverse effects on patients, such as pain and gait disturbance, as well as long term development of post-traumatic arthritis. This paper seeks to highlight some of the options for surgical management of malunions and detail the strategies and approaches used to manage these complicated cases. *Materials and Methods:* An exhaustive search was conducted on PubMed using the key search terms “Tibial” OR “Tibia” AND “Malunion” to be included in the title. Exclusions to the search included any article with patients aged < 18 years, any nonhuman subjects, and any article not published or translated into English. *Results*: A systematic review of the literature revealed 26 articles encompassing 242 patients who had undergone surgical correction for tibia malunion. A total of 19 patients suffered from complications. Methods of treatment included osteotomies, with plate and screws, external fixator, angled blade plate, intramedullary nails, Ilizarov fixator, Taylor Spatial Frame, Precise nail, and total knee arthroplasty. Restoring alignment and the articular surface led to overwhelmingly positive patient outcomes. *Conclusions*: Tibial malunions take many forms, and as such, there are many approaches to correcting deformities. The literature supports the following radiological parameters to diagnose tibial malunion: 5–10 degrees angulation, 1–2 cm shortening, 10–15 degrees internal rotation, and 10–20 degrees external rotation. Surgical plans should be customized to each individual patient, as there are many approaches to tibial malunion that have been shown to be successful in delivering excellent clinical outcomes.

## 1. Introduction

A malunion is broadly defined as a fracture that has healed in a clinically unacceptable position, often resulting in deformity or dysfunction [1]. Malunions of the tibia have the potential to have significant short- and long-term effects on the individual’s biomechanics, cosmesis, and ultimately quality of life [2].

The literature tells us that there is a certain degree of radiological deformity that can be accepted when treating tibia fractures. For the tibial plateau, an initial varus–valgus malalignment of >5 degrees or a joint line incongruity > 1.5 mm makes post-traumatic arthritis more likely. For the tibial shaft, Sarmiento stated that unacceptable parameters include any varus–valgus or anterior–posterior angulation greater than 5 degrees, 15 degrees of internal rotation (IR), 20 degrees of external rotation (ER), shortening > 1 cm, or greater than 50% displacement of any segment about a fracture site [1]. 

The incidence of malunion after tibia fracture treatment after casting and functional bracing has been 0 to 68% [1,2,3,4,5,6,7,8,9,10,11,12,13,14,15,16,17,18,19,20,21,22,23,24,25,26,27,28,29,30,31,32,33,34,35,36,37,38,39] and as high as 20% after intramedullary nailing in distal tibial fractures [33].

Symptomatic malunion from tibia fracture is variable. Several authors have reported that patients with malunions up to and sometimes more than 10 degrees in any plane are asymptomatic [38,39]. Milner et al., (2002) reported on 47 patients with ≥5 degrees of coronal angulation followed up to 30–43 years after suffering a tibia fracture and felt that malunion did not cause osteoarthritis of the knee or subtalar joint [39].

There is a consensus on what is considered a radiographic malunion of the tibia, but it is still debated what the criteria should be to correct a malunited tibia in an asymptomatic patient, especially if many patients with deformity are reported to be asymptomatic in the long term. It seems like any deformity that falls below the values stipulated by our parameters (5–10 degrees angulation, 1–2 cm shortening, 10–15 degrees IR and 10–20 degrees ER) [1,2,3,4,5,6,7,8,9,10,11,12,13,14,15,16,17,18,19,20,21,22,23,24,25,26,27,28,29,30,31,32,33,34,35,36,37,38,39,40,41] is tolerated and deformity around our parameters may be tolerated but more severe deformities are likely not well-tolerated and should be corrected [1,2,3,4,5,6,7,8,9,10,11,12,13,14,15,16,17,18,19,20,21,22,23,24,25,26,27,28,29,30,31,32,33,34,35,36,37,38,39]. Corrections are thus made for (1) a painful functional deficit, (2) an unacceptable cosmetic appearance, or (3) a high risk for degenerative osteoarthritis [2]. This paper seeks to highlight the various options for surgical management of malunions and detail the common strategies used to manage these complicated cases. We will also catalog what deformities surgeons have corrected to get a feel of what was unacceptable to patients.

For the purpose of this paper, the tibia will be divided into three segments: proximal, shaft, and distal. For each segment, we will explore the demographics of patients that suffer malunions, the existing methods of surgical management of malunions, and ultimately the outcomes of these patients.

## 2. Materials and Methods

**Figure 1:**
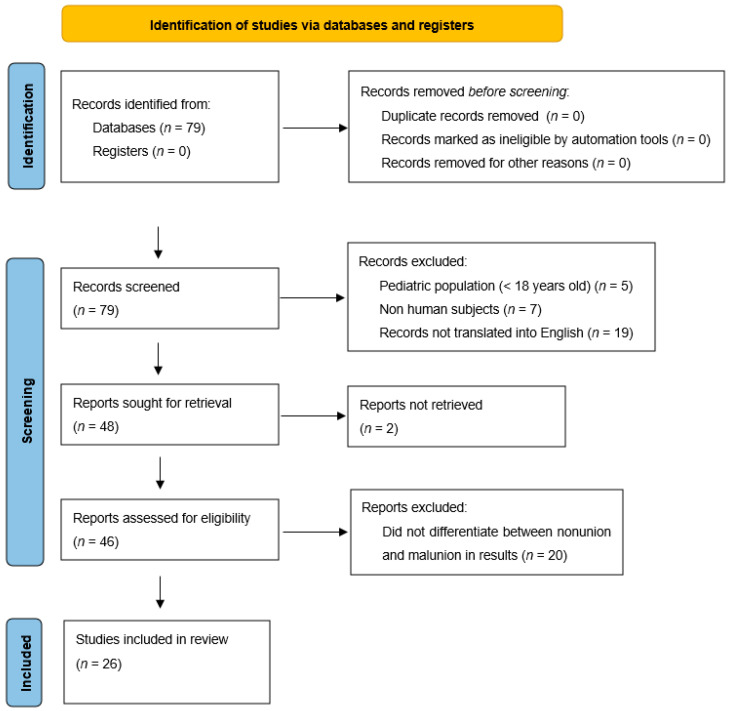
Flowchart displaying methodology for literature review [3].

MEDLINE journals were searched using the PubMed Database to identify relevant studies pertaining to tibia malunion. Key search terms were “Tibial” OR “Tibia” AND “Malunion” to be included in the title. This revealed 79 papers. Exclusions to the search then included any article with patients aged < 18 years, any nonhuman subjects, and any article not published or translated into English. This reduced the number of available papers to 48. We then excluded papers that did not explicitly differentiate between malunion and nonunion. The total number of articles meeting these criteria summated to 28. A total of 2 of these 28 papers could not be accessed, leaving the total count of included studies at 26. Preferred Reporting Items for Systematic Reviews and Meta Analysis (PRISMA) guidelines were used as shown in Figure 1. The data extracted from these studies include the number of patients, area of the tibial malunion, degree of deformity, surgical treatments, amount of correction, and clinical outcomes.

## 3. Results

### 3.1. Patient Evaluation

The patient’s history should focus on preinjury level of activity, their current level of activity, pain level, current perception of their deformity, and disfunction. The physical exam can include an assessment of gait, range of motion/stability of joints, motor strength, limb length discrepancy, neurovascular status, and soft tissue coverage. Before performing any surgical procedures, prior operative reports should be investigated to elucidate approach, exposure, and complications. The patient’s expectations for surgery should also be assessed.

Radiological assessment includes updated radiographs (full-length standing, AP and lateral views of the knee, tibia/fibula, and ankle) and CT scans for rotational alignment. For surgeries involving an articular surface, 2D and 3D CTs should be obtained to further guide the operative plan. An MRI can be shed light on ligamentous and meniscal pathology. To supplement this imaging, it is important to acquire corresponding imaging of the contralateral side to assess the patient’s healthy height, version, and rotation. This way, one can identify the coronal, sagittal, rotational malalignment, and leg length deformity of the limb prior to embarking on any surgical plan for correction [42].

### 3.2. Tibial Plateau Malunion

#### 3.2.1. Demographics and Complications

Fractures of the tibial plateau follow a bimodal distribution, with younger individuals suffering from high-impact mechanisms and elderly individuals suffering from lower-energy mechanisms. Proper fixation of these fractures has an immense impact on the long-term outcome of the patient, as the knee joint is an essential weight-bearing surface. Both patients that undergo conservative treatment and those that undergo primary fixation are susceptible to malunion according to one study of 128 patients [4]. Malunions of the tibial plateau can be classified based on location, geometry, severity, and progression [5]. Patients complain about pain, limp, valgus deformity, and the knee giving out. The decision to repair a malunion of the tibial plateau should be made early on and should be based on patient’s symptomology and biomechanics. In elderly patients, the decision may be made to proceed with total knee arthroplasty [6]. The goals of the surgery are to restore length, alignment, rotation, and anatomic reduction of the articular surface to allow a patient to return to full activity.

#### 3.2.2. Lateral Plateau

There were seven studies found in the literature search which described the treatment of patients with lateral tibial plateau malunion. The two main problems identified were depression of the lateral plateau and valgus deformity. The authors described approaches to visualize the depressed fragments, osteotomies to elevate the depressed fragments, and osteotomies to correct the valgus. They described either proximal tibial osteotomy above the tibial tubercle or lateral tibial plateau osteotomy to correct the valgus malunion. All authors used bone grafts to support the depression and cortical cancellous grafts to support an opening wedge followed by lateral plating.

Marti and Kerkhoffs [8,9] performed oblique osteotomies on 23 patients with lateral tibial plateau depression and valgus malunion of the proximal tibia. Their indications for surgery included: a valgus malalignment on the standing AP radiograph (8 to 20 degrees); and depression of the lateral tibial plateau of 3–21 mm.

The authors utilized a lateral approach and a lateral tibial oblique osteotomy. Exposure is from a lateral parapatellar approach with osteotomy of Gerdes tubercle to visualize the anterior 60% of the plateau, and if a more posterolateral view is required, osteotomy of the fibular head can be performed as well. They described a varus creating opening-wedge osteotomy of the lateral tibial plateau above the tibial tubercle based on a medial hinge, which can be biplanar. The depressed tibial plateau segment is osteotomized through the extra-articular window of the opening wedge from below, elevating it with a tamp to recreate the articular surface. After correction of the patient’s alignment, the osteotomy is filled with bone graft and the depressed segment as well with corticocancellous wedges supported with a lateral buttress plate [7,8].

They achieved an improvement of the mean tibiofemoral angle from 13 to 4.4 degrees, an improvement of the mean lateral depression from 7.7 mm to 1.7 mm. According to the Insall et al. scoring system [9], 20 of the patients had an excellent or good outcome [8].

Yang et al. used CT-generated 3D-printed models to plan the osteotomy cuts for seven patients who suffered posterolateral tibial plateau malunions. The patients had depressed fragments between 4 and 12 mm, at an average of 9.4 mm. They used the 3D-printed model to determine the length and depth of their osteotomy cuts, which were successfully reproduced intraoperatively [10]. Their approach to the lateral tibial plateau was similar to that of Marti and Kerkhoffs [8,9], but their osteotomy was an open-door osteotomy (hinge) of the lateral tibial plateau. They then osteotomized the depressed segment away from the lateral open door and elevated it with a tamp. The defect was filled with graft, the open door closed, and a lateral plate used to buttress the osteotomy.

By the 12-month follow up, all seven patients had achieved fracture union. At this time, the Rasmussen anatomy score as well as the function score had improved for all seven patients (improving from 8.3 to 16.9 and from 15.9 to 26, respectively). In addition, the plateau collapse was less than 1 mm in all seven patients [11].

Mastrokalos et al. [11], Kfuri/Schatzker [12], Furnstahl et al. [13], and Van Nielen et al. [14] described corrections of depressed and valgus lateral tibial plateau malunions by a lateral approach (each in single cases), an open-book osteotomy of the lateral cortex, similar to Yang et al. [11]. They were able to correct deformities of 10-to-20-degree valgus with a depression of 3.6 to 15 mm to normal alignment and correct the depression. Their patients regained full range of motion, were pain-free, and returned to normal activity. Furnstahl [13] used computer-assisted planning with 3D modeling and cutting guides in his report and also corrected a sagittal deformity.

#### 3.2.3. Medial Plateau

There were five reports of medial tibial plateau malunion correction, all single case reports. The difference in these cases, concerning the lateral plateau, is that none of these authors reported a depression in the plateau. All had malalignment and varus angulation of the whole plateau [12,13,15,16,17]. The varus angulation was reported as 7.8 degrees, 9 degrees, 9 degrees, 15 degrees, and 25 degrees. A sagittal flexion deformity was also reported in three cases of 2.6, 27, and 29 degrees. Patients complained of knee pain, effusion, a knee thrust, and limited range of motion. Patients were evaluated with long-leg standing X-rays, CT scans with reconstruction, and, in two reports [14,16], 3D modeling prior to the osteotomy. Several of the authors also recommended an MRI for evaluation of cartilage damage.

Corrections were calculated by using standing films, and each case involved an opening-wedge osteotomy through a medial parapatellar approach. The medial plateau was osteotomized obliquely with a significant bone block, and the cut was made either to the tibial eminence or just medial to it. The coronal and sagittal plane deformity was corrected, the void filled with corticocancellous graft, and the construct supported with a medial plate as was first described by Saengnipanthkul in 2012 [18]. In one paper, due to the late presentation, the osteotomy was followed by a knee replacement [19]. Patients reported good to excellent outcomes at 5 months [13], 6 months [16], 12 months [14], and at 2 years [18] with resolution of pain, return of range of motion, and normal gait.

**Figure 2:**
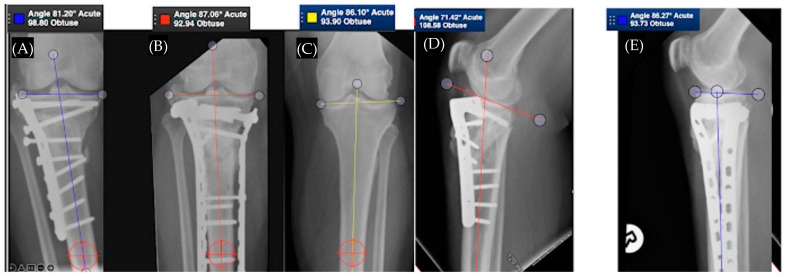
Part (**A**,**D**) show a proximal tibia extra-articular malunion in 6 degrees of varus and 24 degrees in procurvatum. Part (**B**,**E**) demonstrate patient after proximal tibial subtubercular osteotomy and dual plating, with the alignment restored. Part (**C**) depicts the contralateral limb.

**Figure 3:**
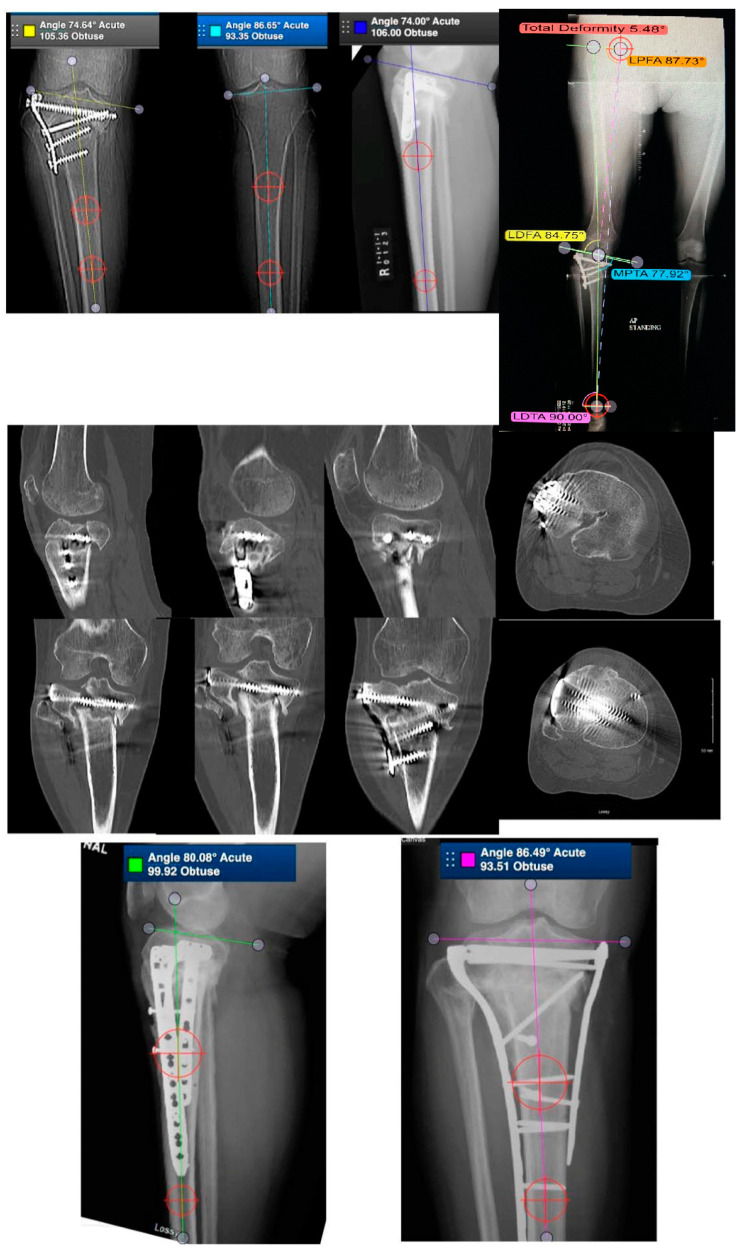
Case of a 54-year-old female with intra-articular malunion of the proximal tibia with 5 mm of lateral depression and 13 degrees of varus deformity (first row of images). CT scan is shown in the second row of images. Patient was treated with a lateral parapatellar approach with a lateral tibial tubercle osteotomy and a trap door osteotomy. The depressed fragments were elevated followed by an opening-wedge tibial osteotomy. Medial and lateral plates were applied to hold the reduction (third row of images).

Figure 2 demonstrates a case of proximal tibia malunion treated with osteotomy and dual plating. Figure 3 demonstrates a case of proximal tibia malunion treated with intra-articular osteotomy.

#### 3.2.4. Posteromedial Plateau

Kfuri and Schatzker described a patient who suffered varus malalignment 18 months after conservative management of a Schatzker IV fracture. The deformity showed a 25-degree varus and 5 mm depression. A posterior approach was used to remove fibrous tissue and mobilize the malunited fragment. Joint congruity was restored, and the fixation was secured with a posterior buttress plate and lag screws.

The patient regained full range of motion and return to normal work activity [12].

#### 3.2.5. Bicondylar Plateau

There was one large series of correction of varus deformities after bicondylar tibial plateau fractures by Wu et al. [19]. They described 28 patients who had previously suffered plateau fractures in motor vehicle accidents and were treated with only lateral buttress plating that had fallen into varying degrees of varus angulation. Fourteen of these patients had fractures that healed in this orientation, classifying them as a malunion, while fourteen had nonunions [18]. These patients were not described with remaining depression of the lateral tibial plateau as that had been successfully corrected with the original surgery.

The surgical technique began with a midshaft fibular osteotomy. A transverse tibial osteotomy distal to the tibial tubercle was utilized, and alignment was manually restored using the medial malleolus and medial edge of the knee. Following realignment, a 95-degree angled blade plate was placed and secured medially to secure the new alignment. The superior end of the blade was inserted aiming at the upper margin of the fibular head. The medial void was filled with cancellous bone graft [18].

A total of 3 patients were lost to follow up, but in the remaining 25 patients, all experienced fracture healing. The proximal medial tibial angle improved from an average of 72 degrees preoperatively to 88 degrees postoperatively upon the most recent follow up. Knee function in all 25 patients preoperatively was unsatisfactory, but 22 of these patients became satisfactory upon the most recent follow up. The other three patients suffered knee pain and less than 90 degrees of flexion at the knee, making their result unsatisfactory [18].

A different problem of a comminuted bicondylar posterior tibial plateau with malreduction was addressed by Van Nielen et al. [15]. The authors utilized the prior posterior approach and restored the extra-articular anatomy but were unable to perfectly restore the articular surface. The articular fragment was removed and preserved so that a CT scan could be obtained to plan for a second revision surgery and allow the authors to restore the patient’s articular surface. For the second revision, an anterolateral approach was utilized. The authors again utilized five osteotomies: the first was a fibular midshaft osteotomy, followed by a Gerdy’s osteotomy, and finally a fibular head osteotomy. Extra-articular tibial osteotomy and distraction restored alignment, while an intra-articular osteotomy was performed so that the previously removed articular piece could be replaced and aligned before bone grafting and posterior and lateral plating [14].

At two years, the patient was able to participate in all activities, was painless with normal range of motion and alignment, and had no arthritic changes [14].

#### 3.2.6. Plateau Widening

Kfuri and Schatzker described a patient who suffered continuing knee pain and valgus malalignment after failed surgical fixation of a Schatzker IV fracture. It was determined that the plateau was widened. In response, the authors overlayed standing radiographs of each leg, calculated the amount of resection required, and proceeded to perform an intra-articular closing-wedge resection osteotomy to remove the excess bone and fibrous tissue.

The patient had normal alignment, full range of motion, and was pain-free at six months [12]. Table 1 and Table 2 describe cases from the studies reviewed regarding tibial plateau malunion.

### 3.3. Tibial Shaft Malunion

#### Demographics and Complications

A tibial shaft fracture is common in younger patients and is usually caused by high-energy trauma and frequently associated with significant soft tissue damage that complicates surgical treatment. Elderly patients suffer tibial shaft fractures from both high-and low-energy mechanisms. Treatment methodology for tibial shaft fractures is guided by the nature of the fracture, the soft tissue injury, and patient factors. Treatment includes reduction and casting, intramedullary nailing, plating, and external fixators [19]. With reduction and casting, a 3–50% malunion rate has been reported [19], and a malunion rate up to 20% has been found in tibial nailing, especially for distal fractures [20].

It is imperative to determine what the true deformity is in a malunion. For the tibial shaft, this will include AP and lateral views, long-leg standing X-rays with sizers to make accurate calculations and CT scans for rotational assessment. It may also include oblique films of the tibial shaft, as often a uniplanar angular deformity can be mistaken for a biplanar deformity if one takes only straight AP and lateral X-rays [1]. Many authors, prior to the year 2000, reported using only X-rays for angular deformity and even clinical exam for rotational alignment [22,23,25]. The use of long-leg alignment views was mentioned by Paley in 1990 [21]. Wu [22] calculated rotational deformities clinically, but by the early 2000s, CT scans were commonly used to measure rotation as described by Murphy in 1987 [23].

Single-plane deformities can be corrected by opening-wedge or closing-wedge osteotomies depending on if the limb is short or long. These can be in the sagittal or coronal plane. The best option is to perform the osteotomy at the site of the deformity or the center of rotation for the angulation (CORA). Biplanar osteotomies have been reported to heal faster and to be more stable during the healing process than uniplanar deformities. In the past, authors have used osteotomies remote from the deformity when soft tissue at the malunion site made it difficult to work there. Graehl et al. [24] corrected tibial shaft deformities of average 15-degree varus in a single plane (six patients). Their strategy was a supramalleolar osteotomy remote from the malunion fixed with a plate or external fixator. This would create a double deformity to achieve their realignment. Fibular osteotomy was also performed.

The final average angulation in the coronal plane for the eight patients was 0 degrees (mean correction of 14 degrees), while the sagittal angulation was 8 degrees of recurvatum. Six of the seven patients with preoperative ankle pain had decreased pain and limping, and this pain disappeared for seven of eight patients [24].

Multiplane deformities are tougher to diagnose and correct. Usually, coronal and sagittal deformities can be corrected with a double-plane osteotomy and still be fixed with a plate and screws, external fixator, or intramedullary nail. These also use a fibular osteotomy to aid the correction. Multiplane with rotational deformities can be corrected with osteotomy and fixed with a plate and screws, but it is difficult. Johnson (1987) [20] Mast et al., (1990) [25], and Sanders et al., (1995) [26] have described methods of evaluating and correcting multiplane deformities with angular components. Significant preoperative planning is required to adequately restore length and perform the correct osteotomy in cases of triplanar deformity, as in these patients. Sanders described his technique, beginning with placing two Schanz pins, one proximal to the deformity, parallel to the tibial plateau, and one distal to the deformity, parallel to the tibial plafond. An oblique fibular osteotomy is then performed at the level of the proposed tibial osteotomy. After the deformity is visualized, the femoral distractor is placed on the Schanz pins. The oblique tibial osteotomy is then performed perpendicular to the plane of maximum deformity as was preoperatively planned. The distractor can then be used to correct length and alignment. Once the surgeon is satisfied with the length and alignment, the fixation is secured with a lag screw perpendicular to the osteotomy and a compression plate is used as a neutralization plate. Finally, a bone graft from the osteotomy is used to fill in any gaps [26]. All three authors reported good results and used plates and screws to fix their osteotomies. They were able to correct LLD of up to 2 cm with slides of the osteotomies or just with the coronal and sagittal correction.

Sangeorzan et al. [27] described a mathematical methodology for determining the exact deformity in three planes and using mathematical equations to make a single tibial osteotomy that will correct the patient’s deformity. The approach uses vector trigonometry and significant amounts of planning to simplify the actual surgical approach. The tibia is osteotomized at the predetermined location and angle and then rotated and fixed into the correct location and fixed with two drill bits. A plate is then used to secure the fixation.

In all cases, the patients’ angular deformities were corrected. One patient developed deep infection, was successfully treated, and had a positive outcome. Four patients with 15 degrees of varus deformity on average, 9.75 degrees of extension, and 1 cm of shortening were corrected to normal measurements. A fibular osteotomy was also performed [27].

Kempf et al. [30] and Mayo and Benirschke [28] described osteotomies for biplanar and rotational deformities using an intramedullary nail with good outcomes for tibial malunions. Using this technique, Wu et al. described 37 cases of tibial shaft malunion and deformity. The patients selected for inclusion had rotational or angular deformities but could not have shortening >2 cm. A total of 95% of the patients had angular deformities, while the other 5% had rotational deformity [22].

A perpendicular tibial osteotomy was performed with respect to the shorter end of the tibia, and both the proximal and distal ends of the osteotomy were anteriorly translated so that they could be sequentially reamed. After aligning the osteotomized tibia, a tibial nail was inserted superiorly and locking screws were placed if rotational instability existed. Finally, cancellous bone collected from the insertion of the nail was used to fill the osteotomy site and the wound was closed. Weight bearing as tolerated was initiated in these patients [22]. Fibular osteotomy was performed concurrently.

Union occurred in these patients at an average of 5.8 months, and 92% of the patients were united at one year. The other 8% of patients were lost to follow up. Two patients suffered deep infection, which resolved with cephalosporin treatment and subsequent removal of hardware. All patients had full range of motion at both the knee and ankle, and no rotational or angular deformities greater than 10 degrees were present. In addition, no patient suffered shortening greater than 2 mm [22].

The correction of tibial malunion using the Ilizarov method was described in the North American literature in the late 1980s and early 1990s for deformities, nonunions, and bone loss [13] (Dr. Ilizarov had described it in the Russian literature well before this time). The concepts of the center of rotation for the angular deformity, percutaneous osteotomy, distraction histogenesis, and the ability to translate bone with a circular apparatus with 6 degrees of freedom changed how deformity correction could be accomplished. This was particularly useful for malunions with greater than 2 cm of leg length discrepancy. Paley et al. [22] described their results in 17 cases with up to 35 degrees of angulation in the coronal and sagittal plane and up to 4 degrees of rotational deformity, and although lengthening capability was present, in this series, the maximum length required to achieve a perfect correction was 2 cm. Complications associated with external fixation such as pin tract infection were common but not problematic, and patients had very good results.

The Taylor Spatial Frame (TSF) is an advance of the Ilizarov external fixator which allows much more mobility of osteotomized fragments, a computerized assessment of deformity, and schedule of instructions to perform the correction over time. It simplifies the planning a surgeon must perform to assess and correct a deformity as it is carried out by a computer program and makes gradual rotational correction easier. It still uses percutaneous distraction histogenesis as originally described by Ilizarov.

Feldman et al. described 11 patients who suffered post-traumatic malunions of the tibial shaft, along with seven patient who suffered nonunions. CT imaging of the affected extremity was used to develop a schedule for the adjustment of the TSF, a specialized circular external fixator that uses stress to drive translation, rotation, and angulation across a patient’s deformity site. Pins were placed above and below the deformity site and connected to the main frame of the TSF. A computer program was utilized to determine the length of time over which the correction should be performed [29]. In their series, the average preoperative coronal and sagittal angulation improved from 11.7 degrees to 1.4 degrees and 10.3 degrees to 0.9 degrees, respectively. The shortening of the tibia improved from 6.8 mm to 0.4 mm. The coronal and sagittal translation improved from 4.9 mm to 0.8 mm and 2.6 mm to 0.7 mm, respectively, following frame application. Range of motion was limited for only two patients who suffered fixed knee flexion deformities of 10 and 15 degrees. Fifteen patients returned to their preinjury level of activity. One patient failed treatment and fell into varus angulation due to nonunion and premature removal of the Taylor Spatial Frame. Three patients suffered superficial pin site infections [29].

A further and less complicated but very effective technique for long bone malunion was described by Russel et al., the clam shell osteotomy [30]. The clamshell osteotomy was designed to treat diaphyseal malunion about the femur and tibia. The osteotomy allows for both translational and angular correction of a malunited diaphyseal segment. Because of this, it is generalizable to virtually all diaphyseal deformities, regardless of type. Their clam shell osteotomy technique uses an intramedullary nail and two transverse osteotomies connected with a longitudinal osteotomy in the coronal plane. The osteotomy was originally designed to be used with an intramedullary rod as the form of stabilization. The intramedullary rod simplifies the deformity correction by acting as a template to restore the axial, coronal, and sagittal plane anatomic axes. The correction is matched to the contralateral limb with intraoperative fluoroscopic images of length and rotation. In addition, lengthening of up to 3 cm can be achieved in the tibia if necessary.

**Figure 4:**
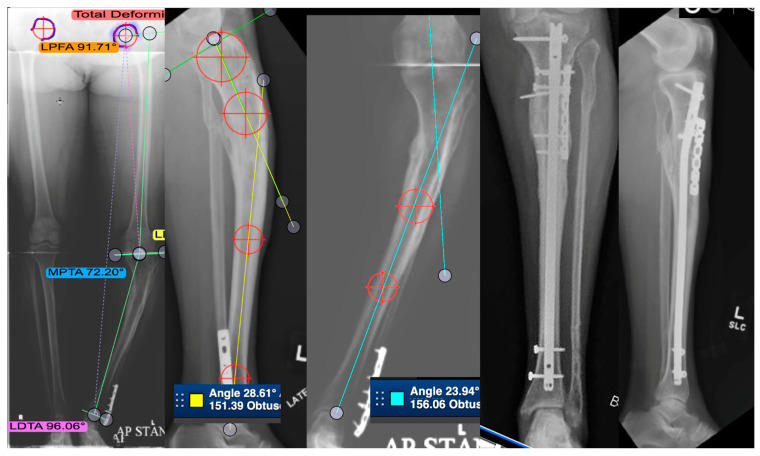
This is a case of a 53-year-old patient with post-traumatic tibial deformity complaining of knee and ankle pain. The deformity was assessed on long-leg standing alignment views, compared to the other tibia in AP and lateral views. CT scan revealed very little rotational deformity. An osteotomy was planned at the center of rotation and angulation (CORA), which was close to the same location on both AP and lateral. This is a single-plane deformity, which has been measured on both the AP and lateral images. The right two images show the patient post-transverse opening-wedge osteotomy fixed with an intramedullary nail and a plate. Healed in good alignment.

Finally, the introduction of lengthening nails, which allow the correction of angular deformity, rotation, stabilization with an intramedullary nail, and the capability of lengthening 6 to 8 cm, has basically allowed the correction of all deformity without the use of a circular Ilizarov-based external fixator. The results for malunion correction are just being reported but are very promising [31]. Figure 4 describes a patient with post-traumatic tibial shaft deformity. Table 3 and Table 4 describe cases from the studies reviewed regarding tibial shaft malunion.

### 3.4. Distal Tibial Malunion

#### 3.4.1. Demographics and Complications

Pilon and distal tibial metaphyseal fractures are usually caused by high-energy trauma in younger patients, in males more than females. Elderly patients suffer distal tibial fractures from both high- and low-energy mechanisms. Treatment methodology for tibial shaft fractures is guided by the nature of the fracture, the soft tissue injury, and patient factors. These fractures progress to malunion in 16.2% of cases [32].

#### 3.4.2. Patients, Surgical Fixation, and Outcomes

Kane and Raiken retrospectively focused on patients with ipsilateral tibial malunion and ankle arthritis who had undergone single-stage revision for the deformity. Twenty-five such patients underwent tibio-talo-calcaneal nailing. Before surgery, most patients had a varus and recurvatum deformity. It was hypothesized that patients with subtalar involvement would benefit from fusion of that joint as opposed to ankle arthrodesis [33].

Careful preoperative planning was needed to establish the center of rotation and to plan the cuts needed for proper alignment. The preferred method of making osteotomy cuts was using fluoroscopy to insert K wires along the preoperatively planned cut angles, followed by osteotome cuts. A retrograde intramedullary nail was inserted through the plantar calcaneus surface, through the talus and into the tibia, at least 5 cm past the tibial deformity [33].

Twenty-four patients were available at 6.5 years, at which time all fusion and osteotomy sites were determined to have healed. On average, VAS scores improved from 8.3 to 2.8, and AOFAS-AH (American Orthopedic Foot and Ankle Society) scores improved from 43 to 76 (88 in the adjusted AOFAS-AH score accounting for the loss of sagittal motion because of the fusion). The average sagittal and coronal plane deformity improved from 26 degrees to neutral and 21 degrees to neutral, respectively. Eighty-four% of patients were extremely satisfied with their surgery, 12% were satisfied, and 4% were unsatisfied. No patients developed tarsal joint or midfoot arthritis at the time of follow up [33].

Shoenleber described eight patients who suffered distal tibial hypertrophic nonunion or early malunion with unacceptable varus–valgus alignment (> 5 degrees) or anterior–posterior alignment (>10 degrees) and an intact articular surface. All identified patients had undergone prior fixation. Presence of callus was identified radiographically and required for inclusion in the study [34].

To correct the malalignment, three patients were placed in an Ilazarov-type fixator and five were placed in a Taylor Spatial Frame. Each of these external-ring-type fixators utilized gradual opening-wedge correction and lengthening to correct the patient’s alignment and length simultaneously. The struts were adjusted to stress the bone based on CT-guided computer calculations. In certain cases, percutaneous osteotomies were utilized to allow great motion of the tibia during the correction phase [34].

The primary outcome for this study was tibial union, which was achieved in all eight cases. Secondarily, a patient’s result was deemed to be excellent if both their varus–valgus and anterior–posterior malalignment were corrected to within 5 degrees of the normal range, acceptable if one but not the other was corrected to within the normal range, and poor if neither had been corrected. Alignment at the time of fixator removal was deemed excellent in five cases, and acceptable in three. Coronal plane alignment was within normal limits in seven patients while sagittal alignment was within normal limits for six patients. There were two complications during treatment, including a broken olive wire and a pin site infection [34].

Nehme et al. described one case of distal tibia pilon malunion that was treated arthroscopically. The patient initially had 6 weeks of treatment via external fixator but had diastasis and step off at the medial malleolus [35].

The patient was positioned, and anteromedial and anterolateral portals about the ankle were created. Initially, the malunited tibia did not move when probed, so the surgeon cleaned the fracture site, which freed the malunited fragment. Percutaneous forceps were used to anatomically align the fragment, which was then secured with self-tapping screws and two percutaneous pins [35]. At 18 months, the patient had adequate bone healing, no pain while walking, and an AOFAS-AH score of 100 [35].

**Figure 5:**
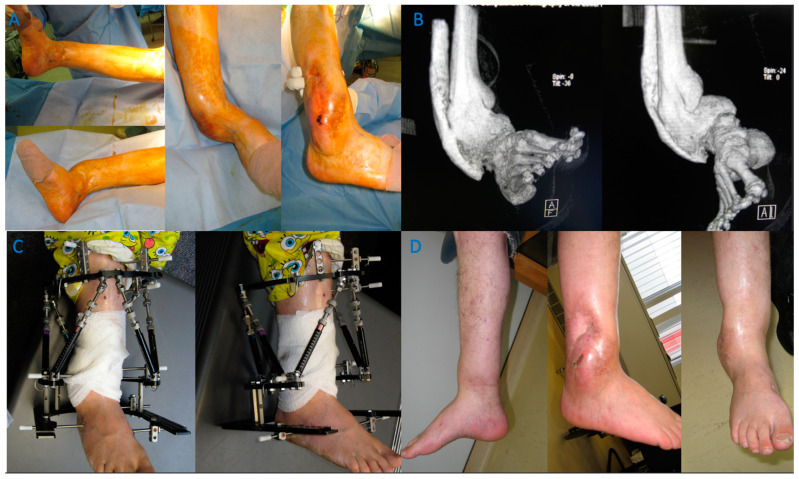
A case of a severe distal tibia deformity with obvious soft tissue damage on the lateral border of the deformity. Conventional X-rays were unable to fully assess or define the deformity that can be seen on a 3D reconstruction CT scan (**B**). Image (**A**) depicts a gross image. An osteotomy and fixation with a Tayler Spatial Frame (**C**) were used with a residual calculation to slowly correct the deformity. Slow correction would help protect the soft tissue envelope on the lateral border and stretch the medial soft tissues. Correction has resulted in a plantigrade foot with motion of the ankle and a healed tibia (**D**).

Surgery to correct intra-articular deformities of pilon fractures are rare because they usually have significant cartilage damage and go on to painful post-traumatic arthritis with malpositioning of the hindfoot and resultant functional disability. Rammelt and Zwipp stated that most of these will need corrective ankle arthrodesis. In their series, they performed 14 cases of pilon malunion that presented early—within 3 months and with intact cartilage, sufficient bone quality, and residual function in a reliable patient. Osteotomies are planned, then carefully thought out and accomplished with thin osteotomes and backfilled with a bone graft fixed with plates and can often give decent results at 5 years [36]. Figure 5 demonstrates a case of a patient with distal tibia deformity and poor soft tissue quality. Table 5 and Table 6 describe cases from the studies reviewed regarding distal tibia malunion.

## 4. Discussion

A malunion is broadly defined as a fracture that has healed in a clinically unacceptable position that is causing the patient dysfunction or disfigurement from deformity. The osseous deformity can be shortening, lengthening, abnormal rotation, or angulation [27]. Radiological accepted parameters to describe malunion are 5–10-degree angulation, 1–2 cm shortening, 10–15-degree IR, and 10–20-degree ER [1,2,3,4,5,6,7,8,9,10,11,12,13,14,15,16,17,18,19,20,21,22,23,24,25,26,27,28,29,30,31,32,33,34,35,36,37,38,39,40].

The orthopedic surgeon’s ability to accurately assess a malunion and deformity has improved from using simple X-rays [1,28,36,39] and assessing rotation by exam [28] to CT scans and deformity measuring software [42]. We can now perform long-leg standing films to assess the overall limb alignment and leg length discrepancy [37] as well as any translational deformity [37]. There are programs available when you purchase a custom hexapod fixator that evaluates the deformity for you and programs the progression of deformity correction [20].

Osteotomies have advanced from uniplanar opening and closing wedges, which could correct some angular deformity in a single plane [1,36,39], to being able to correct multiplanar deformities. A transverse opening-wedge osteotomy can correct a rotational and angular deformity [28]. A percutaneous osteotomy performed with a drill and osteotomes can be used to gradually correct deformities in all planes that can be subsequently lengthened [37], and a clamshell osteotomy allows surgeons to correct angular, rotational and length deformities up to 3 cm acutely [38].

Fixation strategies have evolved from casting to plate and screws [1,36,39], and external fixators to circulator external fixator Ilizarov devices, which are computer-evaluated and even programmed to correct deformities [11]. Finally, remote-controlled nails with percutaneous histogenesis are the next generation of corrective devices [31].

There are many biomechanical effects of malunion that must be considered when deciding if a patient will have a positive outcome with conservative management or if they require surgical intervention. For example, the valgus deformity that results from lateral plateau malunion and depression may result in lateral compartment osteoarthritis. If the knee progresses to this point, the patient is likely to have pain in the future and may significantly alter their gait [38]. There may be concomitant ligamentous changes as the medial ligaments stretch and become lax, causing the lateral structures to tighten. This can lead to complications in the future that may present as instability, or more difficulty for any surgeon that attempts a total knee arthroplasty on these patients.

In general, patients who suffer tibia fractures are prone to ipsilateral osteoarthritis when compared to their contralateral side. In addition, individuals with a concomitant rotational or angulated malunion are even more prone to suffer ipsilateral knee osteoarthritis because of their altered biomechanics and the resulting altered joint surface contact. This additive effect is not observed in patients with a length or translation deformity [38,39,40]. Patients with surgically treated tibial plateau fractures suffer ipsilateral knee osteoarthritis at a rate of anywhere from 10 to 30% [7,8,37]. Even if the articular surface is anatomically repaired, the patient is more likely to suffer from post-traumatic arthritis than someone who did not suffer a plateau injury. This risk only rises with malreduction or malunion [7,14]. As with tibial plateau malunions, patients with tibial shaft malunions are more likely to suffer increased degenerative changes at the knee and ankle. This holds true for both midshaft malunions and distal malunions. Van der Schoot et al. and Tarr et al. reported that angular deformity significantly increased the likelihood of a patient experiencing ipsilateral knee and ankle degenerative changes and osteoarthritis [38,41]. Tarr et al. found that more distal deformities led to more unequal contact of the tibiotalar joint, which is likely to lead to uneven wear and ultimately arthritis [31].

In an effort to avoid this outcome, we have presented many different methodologies for the surgical repair of tibial malunion. The studies mentioned above are firm advocates of early intervention for tibial malunion [14]. There are many surgical methods to restore the patient’s alignment, ranging from performing opening- and closing-wedge osteotomies to various nailing procedures to angled blade plating. The approach should be dictated by the patient’s anatomy and prior surgical approaches, as well as the skill and comfort of the surgeon. Use of CT-guided 3D technology may enhance a surgeon’s understanding of malunions, which are typically multiplanar and complicated [10,13]. Ultimately, in all the studies, restoring alignment and the articular surface led to overwhelmingly positive patient outcomes. Most patients who had their anatomy restored went on to be as functional as they were prior to their injury, had low levels of pain, and could walk without pain or difficulty.

There is a variety of surgical options for the treatment of tibia malunion. These include extra-articular open- versus closing-wedge osteotomy, intra-articular osteotomy, intramedullary nailing, plating versus ringed external fixators, total knee arthroplasty. All of these were used alone or in conjunction with one another and with the majority of them having excellent clinical outcomes. There are advantages and disadvantages to all of these treatments. When treating patients using osteotomies and plate fixation, it is important to keep in mind the importance of non-weight-bearing status as noncompliance can lead to a poor outcome [26]. Treating with a nail may allow for immediate weight bearing, but attaining proper correction may be more difficult for the less experienced surgeon. Treating patients in a Taylor Spatial Frame may allow for very precise correction for deformity, but again this does take a certain level of skill to apply and patient compliance to properly apply the correction [29,34]. Total knee arthroplasty also had excellent results when used for a patient who had already developed osteoarthritis; however, it is also still important to correct the patient’s deformity either using the implant or a corrective osteotomy [16].

Although the majority of patients did have excellent clinical outcomes, there were a number of patients who had negative outcomes. One patient treated for plateau malunion, sixteen patients treated for shaft malunion, and two patients treated for distal tibia malunion all suffered from complications. There was a total of 19 patients with complications. A total of 11 patients had complications due to infection [16,22,24,27,34], the majority of these being pin site infections in those patients which had external or ringed external fixators. There were also two of those eleven patients who suffered from deep infection after undergoing intramedullary nailing and one after plate fixation [22,27]. Two patients likely had complications due to noncompliance after undergoing plating [26]. One of these had anterior skin necrosis, and the other had broken hardware. Four patients who underwent fibular osteotomy had nonunion of the fibula [20]. This is an important consideration when performing the fibula osteotomy to obtain proper correction of the tibia. One patient had a broken wire after being treated with a ringed external fixator, and another had varus collapse and nonunion of the osteotomy site after premature removal of the ringed external fixator [26].

Future research should focus on long-term follow ups of patients who have undergone revision surgeries for tibial malunion to detail the proportion of patients that go on to require total knee arthroplasty, reoperation of the affected limb, and long-term complications. It would be helpful for such a study to compare surgical methodologies to determine the most successful approach for each malunion pattern. In addition, further work should be carried out to help establish criteria for determining which patients benefit from revision as compared to patients who would manage fine with mild malunions.

## 5. Limitations

With this study being a systematic review, it is difficult to draw conclusions regarding the efficacy of any one treatment method compared to another. There were also multiple different surgeons from many different institutions performing these procedures. There were no standardized postoperative protocols, which may be leading to various complications. Again, this makes it difficult to draw conclusions regarding the advantages and disadvantage of any one procedure. Furthermore, with a significant number of studies included in our paper being single case reports, there may be a skew towards positive results as negative outcomes may not have been reported in the literature. Finally, with one of our exclusion criteria being papers that were not translated into English, we may be missing data that might provide valuable insight into the management of tibia malunion.

## 6. Conclusions

A tibia malunion is a fracture that has healed in a clinically unacceptable position that is causing the patient dysfunction or disfigurement from deformity. Radiologically accepted parameters of malunion of the tibia are greater than 5–10-degree angulation, 1–2 cm shortening, 10–15-degree IR and 10–20-degree ER. A patient that has deformity that is significantly larger than these parameters will likely be symptomatic. A patient that has deformity that is close to these parameters may or may not be symptomatic. Tibial malunions take many forms, and as such, there are many approaches to correcting deformities. We have described these various approaches and, to some extent, how management of malunion has changed over the last 50 years. While there are overwhelmingly positive results for patients undergoing deformity correction, care should be taken to customize surgical plans to each individual patient.

## Figures and Tables

**Table 1:** Studies involving tibial plateau malunions, sorted by lateral, posterolateral, medial, or bicondylar involvement.

	Study	Patients (#)	Surgical Treatment	Outcomes
Lateral	Marti et al.	21	Oblique osteotomy w/open-wedge osteotomy for lower-limb alignment correction	Increased range of motion (mean 12°); correction of tibial plateau depression
	Kerkhoffs et al.	23	Combined intra-articular and varus opening-wedge osteotomy	Varus deformities and depression were corrected
	Mastrokalos et al.	1	Reconstruction w/open-book osteotomy	Pain-free; angular deformity corrected; restoration of normal joint congruency, and alignment of axis of knee; function was at preinjury level at latest follow up
	Kfuri and Schatzker	1	Intra-articular osteotomy	Pain resolved, range of motion restored, alignment restored
	Furnstahl et al.	1	Computer-assisted corrective osteotomy w/patient-specific guides	Pain improved in all patients
	Van Nielen DL et al.	1	Revision surgery of tibial plateau involving intra/extra articular osteotomy (1); anterolateral osteotomy (1)	Pain was alleviated, and full range of motion was achieved
	Furnstahl et al.	1	Computer-assisted corrective osteotomy w/patient-specific guides	Pain improved in all patients
	Yang Di et al.	7	3D-printing-assisted corrective osteotomy	Statistically significant improvements in anatomy and function achieved
Medial	Pagkalos et al.	1	Opening-wedge, hemi-tibial plateau intra-articular corrective osteotomy	Symmetrical bony anatomy achieved
	Furnstahl et al.	1	Computer-assisted corrective osteotomy w/patient-specific guides	Pain improved in all patients
	Saengnipanthkul	1	Unicondyle high-tibial osteotomy	Restored medial tibial plateau and medial posterior slope
	Kfuri and Schatzker	1	Medial wedge-opening osteotomy	Bone healing, full range of motion, and return to normal activity
	Hosokawa et al.	1	Corrective osteotomy	Pain resolved; ROM preserved
Posteromedial	Kfuri and Schatzker	1	Corrective osteotomy	ROM restored, return to work activities
Bicondylar	Wu CC	14	Transverse subtubercle osteotomy with lateral open-wedge medial blade plate	Varus deformities were corrected
	Van Nielen DL et al.	1	Revision surgery of tibial plateau involving intra/extra articular osteotomy (1); anterolateral osteotomy (1)	Pain was alleviated, and full range of motion was achieved
Plateau Widening	Kfuri and Schatzker	1	Intra-articular closing-wedge osteotomy	Normal alignment, full ROM achieved, pain-free

**Table 2:** Studies involving tibial plateau malunions, sorted by lateral, posterolateral, medial, or bicondylar involvement. Deformities before and after surgical intervention are displayed.

	Study	Patients (#)	Malunion Deformity	Post-Surgical Alignment
Lateral	Marti et al.	21	Valgus deformity < 20 degrees; lateral plateau depression < 20 mm	Mean improvement of tibiofibular angle (8.6 degrees); mean improvement in lateral plateau depression (6 mm)
	Kerkhoffs et al.	23	Mean valgus deformity 13 degrees; mean plateau depression 7.7 mm	Mean tibiofibular angle 4.4 degrees; mean lateral plateau depression 1.7 mm
	Mastrokalos et al.	1	20-degree valgus deformity with >15 mm lateral plateau depression	No angular deformity or plateau depression
	Kfuri and Schatzker	1	5 mm	Normal alignment
	Furnstahl et al.	1	Plateau depression 3.6 mm; coronal deformity 10; sagittal deformity 7.0 degrees; torsional deformity 12.8	Plateau depression 1.1 mm; coronal deformity improved to 0.5 degrees; sagittal deformity improved to 1.8 degrees; torsional deformity improved to 1.2 degrees.
	Van Nielen DL et al.	1	Valgus deformity 8 degrees with posterolateral fragment depression 3 mm	Normal alignment
	Furnstahl et al.	1	Plateau depression 1.3 mm; coronal deformity 2.4 degrees; sagittal deformity 14.9 degrees; torsional deformity 1.8 degrees	Plateau depression improved to 0.2 mm; coronal deformity worsened to 3.5 degrees; sagittal deformity improved to 4.1 degrees; torsional deformity improved to 0.3 degrees
	Yang Di et al.	7	Mean plateau depression of 9.4 mm	Plateau depression <1 mm for all patients
Medial	Pagkalos et al.	1	Plateau depression 15 mm 9 degrees coronal deformity	Normal alignment
	Furnstahl et al.	1	Plateau depression 6.5 mm; coronal deformity 7.8 degrees; sagittal deformity 2.6 degrees; torsional deformity 2.5 degrees	Plateau depression improved to 1.0 mm; coronal deformity improved to 1.5 degrees; sagittal deformity improved to 0.4 degrees; torsional deformity improved to 0.8 degrees.
	Saengnipanthkul	1	15-degree varus deformity; posteromedial slope 29 degrees;	Restored medial tibial plateau and medial posterior slope
	Kfuri and Schatzker	1	25-degree varus 5 mm depression	Normal alignment
	Hosokawa et al.	1	9-degree varus deformity; 27 degree flexion deformity	Total knee arthroplasty
Posteromedial	Kfuri and Schatzker	1	27-degree varus deformity 5 mm depressed	Normal alignment
Bicondylar	Wu CC	14	Varus knees with proximal medial tibial angle avg of 72 degrees	Proximal medial tibial angle improved to avg 88 degrees
	Van Nielson DL et al.		Not stated	Normal alignment
Plateau Widening	Kfuri and Schatzker	1	Not stated	Normal alignment

**Table 3:** Studies involving tibial shaft malunions.

Study	Patients (#)	Surgical Treatment	Outcomes
Graehl et al.	8	Supramalleolar dome osteotomy or closed-wedge osteotomy	Improvement in pain in six patients; decrease in limping for 7 patients; improvement in sagittal plane deformity in all patients; coronal plane improvement in 1 of 3 patients with significant presurgical deformity
Sanders et al.	12	Oblique osteotomy	Correction of sagittal deformity to within 2° of normal; correction in coronal plane within 1° of normal; avg 1.3 cm of lengthening obtained; full range of motion (10); full-weight bearing; and return to previous employment (10); two patients failed initial revision
Kempf et al.	7	Reamed intramedullary nail	Good correction and all healed
Mayo and Benirschke	23	Reamed intramedullary nail	All healed, 1 infection with acceptable alignment
Sangeorzan et al.	4	Single-cut oblique osteotomy	All angular deformities were corrected. One patient developed deep infection, was successfully treated, and had a positive outcome
Wu CC et al.	37	Reamed intramedullary nailing	All patients’ deformities reached <10° angulation and/or rotation and <2 cm shortening; limping gait was corrected
Johnson	7	Multiplane corrective osteotomy	Lower-leg deformity and angulation improved in all patients; 2 patients had improved back pain; ipsilateral knee pain relieved (4); ipsilateral ankle discomfort resolved (2); improved back pain (2); gait improved in all patients; fibula nonunion (4)
LaFrance et al.	1	2 stage: corrective fibula osteotomy followed by revision ACL reconstruction	Instability resolved
Feldman et al.	11	Taylor Spatial Frame	Mean alignment: coronal angulation 1.4°, sagittal plane 0.9°, shortening 4.4 mm, rotation 0.6°
Lahav and DiMaio	1	Opening-wedge osteotomy and total knee arthroplasty	Excellent range of motion and a stable knee to varus and valgus stress. Radiographs at two years illustrated a well-seated TKA and correction of tibial alignment
Paley et al.	17	Ilizarov	Angular > 5° rotation > 15° lld > 1.5 mean 4.5 cm
			All corrected within 1.5 cm lld angular deformity < 5° and rotation < 10°

**Table 4:** Studies involving tibial shaft malunions. Deformities before and after surgical intervention are displayed.

Study	Patients (#)	Malunion Deformity	Post-Surgical Alignment
Graehl et al.	8	Varus deformity 15 degrees; one patient had an anterior sagittal plane deformity of 14 degrees; two had posterior deformities of 30 degrees and 45 degrees	Average coronal plane alignment 0 degrees; sagittal angulation was 8 degrees of recurvatum on average; only 1 of 3 patients with sagittal deformity were in neutral alignment
Mast et al.	17	>8-degree coronal, 5–40-degree sagittal, 10 IR-15 ER, leg length discrepancy 1–2 cm	Healed corrected within 5 degrees
Sanders et al.	12	Mean sagittal plane deformity 13 degrees; mean coronal plane deformity 23 degrees of procurator; average shortening 2.2 cm	Correction of sagittal deformity to within 2° of normal; correction in coronal plane within 1° of normal; avg 1.3 cm of lengthening obtained
Sangeorzan et al.	4	Varus deformity 15 degrees on average; 9.75 degrees of extension on average; 1 cm of shortening	All corrected to within acceptable limits
Wu CC et al.	37	Angular or rotational deformity, but no shortening >2 cm	All patients’ deformities reached <10° angulation and/or rotation and <2 cm shortening
Johnson	7	Varus in six patients averaged 16.6 degrees; shortening averaged 1.29 cm in six patients; posterior bowing averaged 8.2 degrees in five patients.	Lower-leg deformity and angulation improved in all patients
LaFrance et al.	1	15-degree apex anterior; valgus 10 alignment	Normal alignment
Feldman et al.	11	Mean alignment: coronal angulation 11.7°, sagittal plane 10.3°, shortening 6.8 mm, rotation 0.6°	Mean alignment: coronal angulation 1.4°, sagittal plane 0.9°, shortening 4.4 mm, rotation 0.6°
Lahav and Dimaio	1	Varus malalignment at the knee was 12 degrees; 20-degree varus malunion of the tibia	Normal alignment
Paley et al.	17	Angular rotation >5° (avg rotation >15°) leg length discrepancy >1.5 (mean 4.5 cm)	All corrected within 1.5 cm leg length discrepancy, angular deformity < 5°, and rotation < 10°

**Table 5:** Studies involving distal tibial malunions.

Study	Patients (#)	Surgical Treatment	Outcomes
Kane and Raikin	16	Single-stage corrective osteotomy w/tibiotalocalcaneal nailing	VAS * pain scores improved from 8.3 to 2.8; AOFAS ** functional scores improved from 43 to 75; all deformities corrected to neutral alignment
Schoenleber and Hutson Jr.	5	Deformities corrected w/gradual opening wedge osteotomy w/lengthening when indicated	Lower-leg deformity equalized
Nehme et al.	1	Arthroscopy-assisted mobilization w/percutaneous fixation	Patient regained full physical activities; American foot and ankle score of 100

* Visual Analog Scare; ** American Orthopedic Foot and Ankle Society Score.

**Table 6:** Studies involving distal tibial malunions. Deformities before and after surgical intervention are displayed.

Study	Patients (#)	Malunion Deformity	Post-Surgical Alignment
Kane and Raikin	16	Varus and recurvatum combination was the most commonly seen deformity; average sagittal plane malalignment was 26°, and the average coronal plane malalignment was 21°	All deformities corrected to neutral alignment
Schoenleber and Hutson Jr.	5	Varus deformities (8–19 degrees);valgus deformities (16 degrees); apex anterior deformities (2–21 degrees); apex posterior deformity (range, 9–20 degrees).	Lower-leg deformity neutralized to less than 5 degrees angulation in all TSF patients and to less than 5 degrees in one plane and less than 10 degrees in the second with the Ilizarov
Nehme et al.	1	Malunited medial malleolus	Within normal limits
Rammelt and Zwipp	14	Malunited pilon fractures treated with intra-articular osteotomy presented at 3 mo	5-year follow up Excellent 1, good 9, fair 2 poor 2 7 revision surgeries and 2 arthrodesis
